# Towards an Explainable Universal Feature Set for IoT Intrusion Detection

**DOI:** 10.3390/s22155690

**Published:** 2022-07-29

**Authors:** Mohammed M. Alani, Ali Miri

**Affiliations:** 1 Computer Science Department, Toronto Metropolitan University, Toronto, ON M5B 2K3, Canada; ali.miri@ryerson.ca; 2 School of IT Administration and Security, Seneca College of Applied Arts and Technology, Toronto, ON M2J 2X5, Canada

**Keywords:** IoT, intrusion detection, security, dataset, machine-learning

## Abstract

As IoT devices’ adoption grows rapidly, security plays an important role in our daily lives. As part of the effort to counter these security threats in recent years, many IoT intrusion detection datasets were presented, such as TON_IoT, BoT-IoT, and Aposemat IoT-23. These datasets were used to build many machine learning-based IoT intrusion detection models. In this research, we present an explainable and efficient method for selecting the most effective universal features from IoT intrusion detection datasets that can help in producing highly-accurate and efficient machine learning-based intrusion detection systems. The proposed method was applied to TON_IoT, Aposemat IoT-23, and IoT-ID datasets and resulted in the selection of six universal network-flow features. The proposed method was tested and produced a high accuracy of 99.62% with a prediction time reduced by up to 70%. To provide better insight into the operation of the classifier, a Shapley additive explanation was used to explain the selected features and to prove the alignment of the explanation with current attack techniques.

## 1. Introduction

**Figure 1:**
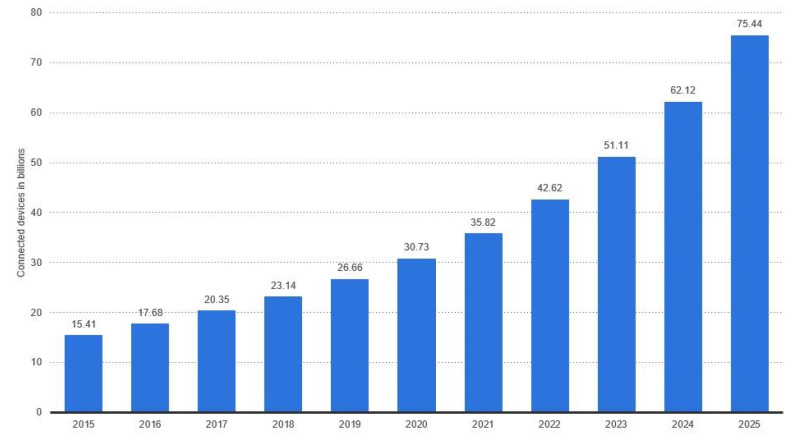
Internet-Connected IoT Devices Growth [1].

The adoption of the Internet of Things (IoT) is rapidly increasing, and IoT devices are becoming more ubiquitous in our daily lives. Figure 1 shows the rapid growth of devices connected to the Internet throughout the years. As shown in the figure, the number of devices is expected to exceed 40 billion by the end of 2022. This rapid growth comes with multiple security problems.

According to [2], the past five years have witnessed dramatic surge in IoT attacks globally. These attacks were driven by the increase in device adoption and the lack of security awareness on the user side. The first half of 2021 witnessed 1.5 billion attacks on IoT devices, compared to 639 million in the second half of 2020 [3]. These attacks mainly focused on the use of the breached IoT devices to steal personal or corporate data, mine cryptocurrencies, and conduct distributed denial of service (DDoS) attacks in which the devices are added to a botnet.

Major IoT security challenges are summarized in the following points:

1. The focus of many IoT manufacturers is around optimal efficiency in production, and overlook security concerns in the devices they produce. According to [4], many manufacturers utilize outdated open-source firmware with many known vulnerabilities without any patching or security testing;
2. On rare occasions that manufacturers issue patches, these patches are usually difficult to apply, and non-technical users face difficulties in applying them and end up not applying them successfully. Most of the used firmware does not support Over-The-Air (OTA) updates, and this makes the pathcing process very challenging and error prone;
3. IoT devices are known to be resource-constrained. The available memory and processing power are usually limited and barely adequate for the devices to do its job.This makes them hard to defend at the host-level;
4. Many IoT device users do not change the default settings. This means that many devices use their default usernames and passwords that can easily be guessed or brute-forced, as in the case of the Mirai botnet [5]. In certain cases, these credentials are hard-coded into the firmware and cannot be changed by users.

The points mentioned above make the case for a network-based defense strategy instead of a host-based one. Network-based intrusion detection offsets the overhead of the detection process to the network border and enables the use of devices with higher processing power, memory, and storage capacities.

Network-based intrusion detection based on machine learning has been the subject of many studies in the past two decades. One major challenge faced by these systems is the lack of a universal feature set that can represent a wide range of attacks, while not being localized to a specific dataset. Different systems followed different ways of selecting the suitable features but were rarely tested for generalization beyond their training datasets.

While machine learning has a great potential in addressing many cybersecurity problems, its adoption is not as rapid as the threats are developing. One major obstacle in the way of adopting machine learning-based cybersecurity solutions is the lack of explainability. Many solutions are built and presented to the cybersecurity society as a black-box solution whose decisions are “mostly” correct. To overcome this obstacle, we present in this work an explainable machine learning solution using Shaply additive explanation (SHAP). This explanation helps in understanding how the model is making its predictions such that they no longer come from a “blackbox”.

### 1.1. Research Contribution

In this research, a feature selection method that is focused on efficiency and implementability is introduced to produces a universal set of features to train and deploy models with higher efficiency while maintaining high accuracy. This paper makes the following contributions:

- Reduce the number of features needed to create a high-accuracy intrusion detection model. The selected features were only six flow-based network features;
- Achieve an accuracy of 99.62% in the testing of the trained machine learning classifier dataset;
- Explain the selected features using SHAP values to provide a better understanding of how the model makes a prediction;
- Create a smaller version of the TON_IoT dataset that can be used in real-life implementations of machine learning-based IoT IDS.

### 1.2. Paper Layout

This paper is divided into nine sections. Section 2 discusses the related previous works to pave the way to Section 3 discussing TON_IoT dataset that was used in our experiments. Section 4 presents the details of the steps taken to prepare the dataset for training and testing. Section 5 explains the proposed feature selection mechanism. Section 6 shows the implementation testing results. Section 7 shows the models explanation using SHAP values, while Section 8 discusses the implementation considerations that need to be considered when deploying the model in a real-life environment, and compares the results to previous works and discusses how the proposed feature reduction produces high accuracy. Section 9 provides our research conclusions and directions for relevant future research.

## 2. Related Works

The use of machine learning in intrusion detection has been area of rigorous research for a long time [6,7]. Intrusion detection in the IoT context was also addressed in many research publications [8,9,10]. Feature selection for IoT intrusion detection has been a challenging task that several research papers have tackled. Selecting a high number of features is generally associated with higher processing overhead, lower efficiency originating from the need to extract more features at the data acquisition stage, and longer time to produce a prediction when the number of inputs is high. On the other hand, a lower number of features reduces the prediction time, reduces the number of features to be extracted, and hence improves efficiency and reduces the processing overhead. Within this section, and for the sake of comparability, we will review papers that specifically address the issue of feature selection.

Desai et al. presented, in 2020, an intrusion and botnet detection system for IoT devices [11]. The proposed system built a multiclass-classifier using supervised learning models with Principal Component Analysis (PCA) for dimensionality reduction. The proposed system used the dataset presented in [12]. Although the proposed system achieved high accuracy of 0.9871 using a Random Forest (RF) classifier, with features reduced to 10 using PCA, the results are considered non-generalizable because the dataset included IP addresses, and MAC addresses of the attack and victim machines. This causes the classifier to suffer from overfitting and perform poorly beyond its training dataset.

Moustafa presented, in 2021, another article discussing the TON_IoT dataset collection mechanisms along with the feature extraction techniques used [13]. Although the paper did not discuss feature reduction explicitly, it discussed scaled feature importance for the network flows part of the dataset, which was reasonably aligned with the findings of our research. The main focus of the paper was presenting a distributed testbed architecture of IoT network that can be used for the evaluation of machine learning-based security applications.

Khan et al. published, in 2021, a paper discussing the detection of attacks on Medical IoT (MIoT) with the use of eXplainable Artificial Intelligence (XAI) [14]. The proposed method reduced the dimensionality using Principal Component Analysis (PCA). The proposed method produced a high accuracy of around 99%. However, the use of PCA negatively impacts the implementation in real life. The main reason is that the number of captured and extracted features will remain the same, while additional preprocessing is to be performed on these features to produce a lower number of features. This impacts the efficiency of the data acquisition and prediction process.

Nimbalkar et al. introduced, in 2021, a study focusing on feature selection for IoT Intrusion Detection Systems (IDS) [15]. The study proposed feature selection using Information Gain (IG) and Gain Ratio (GR) with the top 50% ranked features for the detection of Denial of Service (DoS) and DDoS attacks. The proposed method was evaluated on the IoT-BoT and KDD Cup 1999 datasets, respectively, and provided a higher performance than the original feature set and traditional IDSs on the IoT-BoT and KDD Cup 1999 datasets using 16 and 19 features, respectively.

In 2022, Sarhan et al. proposed a standard feature set for network intrusion detection datasets [16]. The paper focused on general network flow-based intrusion detection including IoT intrusions, as well as other network intrusions. The paper combined four datasets including BoT-IoT, and Ton_IoT, which are IoT-specific datasets. The paper proposes two feature sets; one with 43 features, and a smaller one with 12 features only. The experiments presented in the paper showed that the 43-feature datasets present better performance compared to the 12-feature datasets. The 43-feature version achieved an accuracy of 0.9786 with a prediction time of 8.3 μs.

## 3. The Dataset

The first datasetto be used for feature selection is TON_IoT, which was introduced in 2019 in [17]. The dataset includes data collected from real IoT, and Industrial IoT (IIoT) devices. The sources of data provided heterogeneous data collected from telemetry datasets of IoT and IIoT sensors, operating systems datasets of Windows 7 and 10 as well as Ubuntu 14 and 18 Transport Layer Security (TLS) and network traffic datasets. The dataset was collected from a realistic and large-scale network designed at the Cyber Range and IoT Labs, the School of Engineering and Information technology (SEIT), UNSW Canberra at the Australian Defence Force Academy (ADFA).

The collected datasets were split into three different sets:

1. Raw datasets
  Collected from 10 IoT and IIoT sensors, network capture (pcap), Linux tracing tool dataset, and Windows collectors of the Performance Monitor Tool on Windows 7 and 10 systems;
2. Processed datasets;
3. Train-Test-datasets.

As the focus of this paper is directed towards flow-based intrusion detection, the subset of the dataset that is being used in this research, is the network-based train-test dataset. This dataset contains 461,043 records extracted from network flow features; including 300,000 benign, and 161,043 malicious flows. Table 1 shows a list of categories and attacks captured in this dataset and displaying the numbers of records in each category.

The dataset was created by extracting 44 features from the raw packets to produce the 461,043 network-flow instances. Detailed list of features of the dataset can be found in [17].

The second dataset, named IoT-ID, was presented in [12]. The dataset was created using real IoT devices and consists of 42 network packet capture files (pcap) holding 2,985,994 packets. These packets are divided to 1,756,276 benign, and 1,229,718 malicious packets. These pcap files will be used to extract network-flow information, as our model operates at the network flow level, not at a packet level.

This dataset includes malicious attacks within the following categories:

1. Man-In-The-Middle (MITM) ARP Spoofing;
2. Denial of Service attack (SYN flooding);
3. Mirai botnet (UDP flooding, ACK flooding, HTTP flooding, host discovery, telnet brute-force);
4. Port and Operating System (OS) scanning;
5. Host scanning.

## 4. Preprocessing

### 4.1. Classifier Selection

For the implementation of the proposed feature selection algorithm, we used the Sci-KitLearn machine-learning library in Python. Our experiments included four different machine-learning classifiers listed below:

- Random Forest;
- Logistic Regression (LR);
- Decision Tree (DT);
- Gaussian Naive-Bayes (GNB).

### 4.2. Dataset Observations and Preprocessing Steps for TON_IoT

The dataset underwent detailed examination to find any anomalies that might hinder the training process. Our findings include:

- Missing values were replaced with a ‘-’;
- MITM attack category represented only 0.22% of the dataset;
- Several data fields included non-numerical values, such as source and destination IP addresses, protocol, and service types;
- There were features that logically do not impact the predictions, such as timestamp;
- The “malicious” and “benign” labels are reasonably balanced with 300,000 benign instances, and 161,043 malicious instances;
- The dataset includes features that are host-specific such as the src_ip and dst_ip.

These findings were addressed with the steps shown in Algorithm 1.
**Algorithm 1:** TON_IoT Dataset Proprocessing**Input:** TON_IoT train-test Dataset (461,043 instances, 45 features) **Output:** Balanced Dataset with no missing data (461,043 instance, 37 features) Array←Dataset
In (Array) remove type label In (Array) remove $weird\_notice,weird\_add,weird_{n}ame,ts$ features In (Array) remove src_ip,dst_ip features label-encode nonnumericalfeatures


As shown in Algorithm 1, the first step of preprocessing was to remove the attack type label. The reason behind this removal was that we noticed very large differences between the number of packet flows in MITM attack and other attacks. In addition, the scope of our research was focused on identifying “benign” and “malicious” traffic, not specifying the type of the attack. Hence, we removed the attack type labels to utilize binary classification, with “malicious” and “benign” labels instead.

The next step was to remove features that would have a negative impact on the training process without contributing to the detection process, such as the ts feature carrying a numerical value of the timestamp. Other features such as weird_notice, weird_add, weird_name were removed as well.

In the following steps, we removed IP addresses of source and destination to ensure that the trained model can generalize well beyond its training dataset. In addition, label encoding was performed on the features with the names proto, service, conn_state, dns_query, dns_AA, dns_RD, dns_RA, dns_rejected, ssl_version, ssl_cipher, ssl_resumed, ssl_established, ssl_subject, ssl_issuer, http_method, http_uri, http_user_agent, http_orig_mime_types, http_resp_mime_types as they did not carry numerical values in the original dataset.

As the preprocessing phase was concluded, the resulting dataset had 461,043 instances in total (300,000 benign, and 161,043 malicious). Each instance carrying 37 features.

### 4.3. Observations and Preprocessing Steps for IoT-ID

As two additional datasets were used for validation of feature selection, the preprocessing of these datasets, IoT-ID [12] and Aposemat IoT-23 [18], was performed after the feature selection step in TON_IoT, and hence, it would directly result in a 6-feature dataset.

By examining the information provided with the IoT-ID dataset, we found that the “UDP flooding” sub-category in attack packets is about 949,284 packets, which forms about 77% of the malicious class. To provide proper balance, we randomly removed 809,726 packet from this sub-category to keep the malicious packets at 419,992.

Upon examining the Aposemat IoT-23 dataset, we randomly selected 100,000 instances (50,000 benign, and 50,000 malicious) from the different attack scenarios presented in the dataset.

The following preprocessing steps were performed to prepare the datasets for testing:

1. The original pcap files were split into benign and malicious pcap files according to the information provided with the dataset;
2. The split pcap files were converted into network flows using a tool named Zeek [19]. This tool generates network flow information files, named “conn.log”, in a special format named Zeek logs. We used a Python tool named ParseBroLogs [20] to parse these logs and generate corresponding CSV files;
3. The CSV files were combined into a single dataset containing 20 features, including the 6 feature that were selected in our experiments;
4. The additional features were removed, and the selected 6 features were ordered in a similar order to the one used in TON_IoT dataset;
5. The last preprocessing step was to perform label encoding to the proto and conn_state features using the same encoding that was used in the TON_IoT preprocessing phase.

The preprocessing stage produced a dataset with six features and 198,064 network flows divided into 111,345 malicious and 86,719 benign flows.

## 5. Proposed Feature Selection

This research aims to select the most effective features in IoT introsion detection. This explainable selection of features also focused on reducing the number of features acquired at the data acquisition stage, and not only on dimensionality reduction in machine learning-based model. This research aim means that we cannot rely on statistical dimensionality reduction algorithms such as PCA, Singular Value Decomposition (SVD), and Linear Discriminant Analysis (LDA) [21]. This is due to the fact that these techniques will need to be applied to the raw data captured before being usable to feed into the classifier. Using these techniques, i.e., PCA, LDA, or SVD, can impact the implementation efficiency by consuming additional processing power to preprocess the raw data before sending it to the classifier. In addition, using these algorithms will also mean that a larger number of features will be captured, and preprocessed to produce the required input to the classifier. Our proposed approach will avoid this by using raw features extracted from real-life deployment.

The method that we proposed to perform feature selection in this research was recursive feature elimination (RFE) using feature importance. The summarized steps of RFE are shown in Algorithm 2.


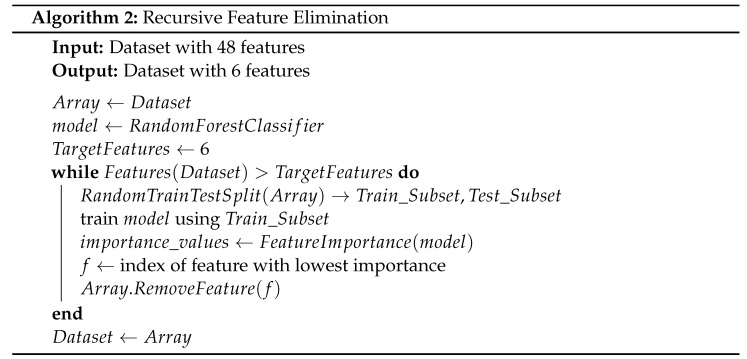
 

As shown in Algorithm 2, the first step is to create an RF classifier to be trained with randomly selected 75% instances from the dataset, and later tested with 25% of the instances. The next step is to calculate the feature importance of features. *Feature importance*, as defined in [22], is the average impurity decrease computed from all decision trees in the forest without assuming linear separability of the data. After calculating the feature importance, the with the lowest score would be eliminated. Then, another round of training and testing is performed, and the feature with the lowest feature importance is then removed. This process is repeated, and $F_{1}$ Score is recorded for each cycle so we can track the model’s performance to prevent it from large drops in performance. Based on this selection method, not only the number of features in the input to the classifier is reduced, but the number of features that need to be captured and extracted is reduced as well. This enables improved efficiency at multiple stages of the system operation such as data acquisition, training, testing, and helps produce further realistic real-life deployment.

As shown in Section 2, Moustafa [13] also used feature importance for feature selection. However, the approach was different. Moustafa’s approach was to calculate the feature importance after training and select the feature with the highest importance as the final features. This approach ignores the possible correlation of these features with the low-importance ones. Our approach of using feature importance was different. Our proposed method utilizes recursive elimination of the feature with the lowest importance. This approach considers the possibility of correlation of features of high importance and low importance. Removing the feature with the lowest importance and then re-training the model might create a different feature importance in other features as they are affected by their existence, and elimination is affected by the lowest importance feature.

## 6. Implementation and Results

### 6.1. Performance Metrics

According to [23], each machine learning based binary classifier produces the following for measures:

1. True Positive (*TP* );
2. True Negative (*TN*);
3. False Positive (*FP*);
4. False Negative (*FN*).

These four metrics, when combined together, generate the confusion matrix.

In our research, the following six performance metrics are used:

1. Accuracy
  (1) $$Accuracy=\frac{TP+TN}{TP+TN+FP+FN}$$
2. Precision
  (2) $$Precision=\frac{TP}{TP+FP}$$
3. Recall
  (3) $$Recall=\frac{TP}{TP+FN}$$
4. $F_{1}$ Score
  (4) $$F_{1}Score=2*\frac{Precision*Recall}{Precision+Recall}.$$
5. Training Time
  The time spent in training the classifier (measured in seconds).
6. Testing Time
  The time spent by the trained classifier to process one input instance and produce a prediction.

### 6.2. Testing Strategy

To ensure that the experiments meet the research goals, a testing strategy was devised. This strategy is explained in the following subsections.

#### 6.2.1. Initial Testing

At the initial testing stage, we created the four classifiers; RF, LR, DT, and GNB. These classifiers were trained using 75% entries of the preprocessed 37-feature TON_IoT dataset that were selected randomly. Then, these four classifiers were tested using the remaining 25% entries of the dataset. The purpose of this testing was to choose the best performing algorithm to use it in the proposed feature selection algorithm.

#### 6.2.2. Post Feature-Selection Testing

Another round of training and testing took place after the feature selection process. The four classifiers were re-trained using randomly selected 75% of entries of the reduced TON_IoT dataset, and were then tested using the remaining 25% entries. The purpose of this testing is to ensure that the system performance did not degrade after selecting a smaller number of features.

#### 6.2.3. 10-Fold Cross-Validation

An important part of our research is ensuring that the selected features are “universal” and that the trained model generalizes well beyond its training dataset. For that purpose, we implemented 10-fold cross-validation. The steps of this algorithm are shown in Algorithm 3.


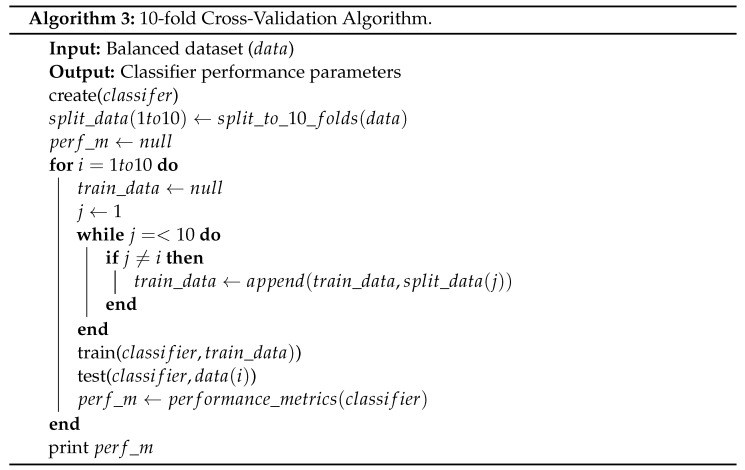
 

Within this validation process, the dataset is randomly split into ten parts. After the split, the model undergoes ten cycles of training and testing. In each cycle, one of the ten subsets is used for testing while the other nine are used for training. By the end of these cycles, all subsets have been used once for testing. The performance metrics of the all of the ten cycles are measured. If these metrics have high variance, then the classifier is suffering from over-fitting and is not generalizing properly within the dataset. If the variance is low, then the the classifier is capable of generalizing well beyond its training dataset, and the mean values of the performance metrics obtained from these 10-folds can be considered reliable results [24].

The purpose of this testing stage, as stated earlier, is to ensure that the classifier is capable of generalizing well beyond its training dataset.

#### 6.2.4. Testing with IoT-ID and Aposemat IoT-23 Datasets

Another step to validate the trained model and the selected features is to test it using two dataset that are different from the one used in training.

#### 6.2.5. Live Attack Testing

Our final testing step is to test the trained model using a live port scanning attack from another machine using nmap tool [25].

### 6.3. Testing Results

#### 6.3.1. Testing before and after Feature Selection

As mentioned in Section 6.2, the first step is to train and test the classifiers using the 37-feature version of the dataset to create a baseline to compare the reduced version to. At the first step, the four classifiers were trained and tested with the preprocessed dataset before feature selection. The next round of training and testing took place after the feature selection process to measure the impact of the selection process on the models’ performance.

**Figure 2:**
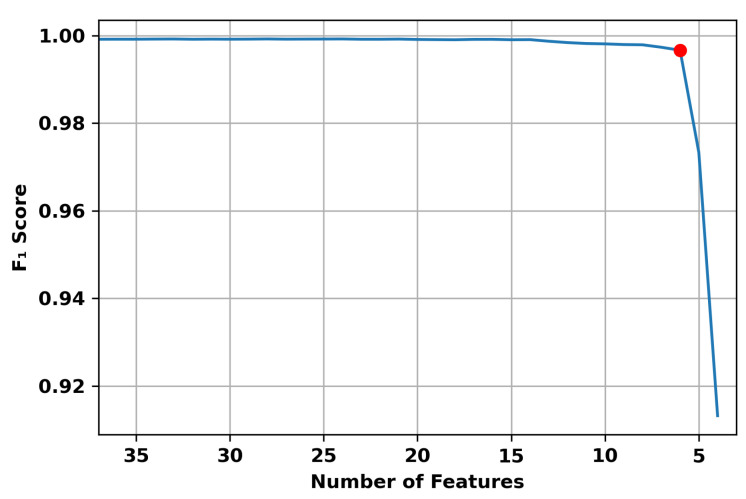
$F_{1}$ Score change with change in number of features.

The recursive elimination of the lowest importance feature was concluded with the selection of six features. These features are dst_port, proto, conn_state, src_pkts, src_ip_bytes, dst_ip_bytes. Figure 2 shows the impact of feature reduction on the $F_{1}$ score of the trained model.

As shown in the figure, the $F_{1}$ Score maintained a high value within the 0.99 region despite the reduction of features until it reached a certain threshold, six features, where the performance started to drop rapidly. Hence, the number of features selected was chosen to be six to prevent the performance from degrading due to further reduction.

Table 2 shows the accuracy and $F_{1}$ score before and after the feature selection process in the form of a comparison. $F_{1}$ Scores shown in the table were calculated as the weighted average $F_{1}$ Score. In addition, we showed the *FP* and *FN* measured in both cases.

As shown in Table 2, all algorithms maintained comparable performance measures while being trained and tested with 37 features, and six features only. In general, RF and DT provided outstanding performance when compared to LR and GNB.

Maintaining high accuracy after the feature selection process, in the case of RF and DT, is an evidence that the feature selection process was successful in achieving the goal of maintaining high accuracy with a noticeably smaller features set.

**Figure 3:**
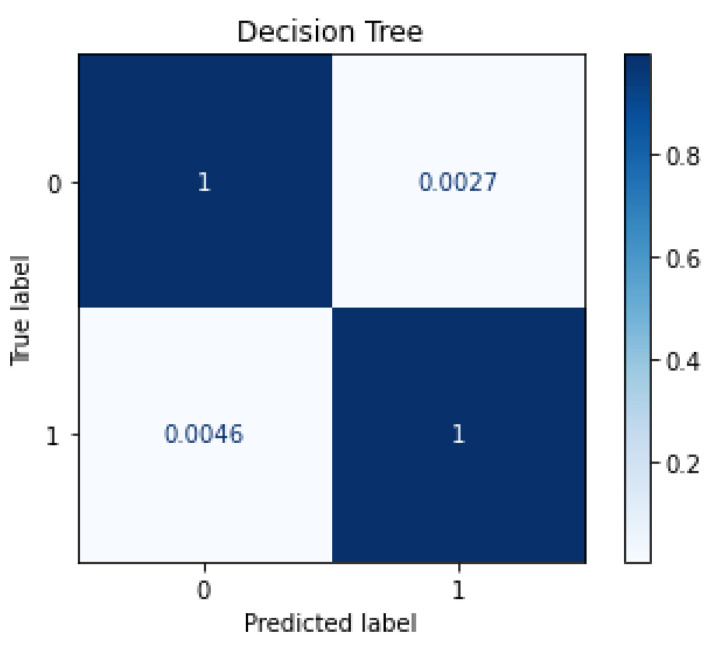
Confusion Matrix Plot of DT Classifier with 6 Features.

While RF achieved a slightly higher accuracy, the testing time achieved by DT, as shown later in Section 8, was about 14 times lower. Figure 3 shows the confusion matrix plot for DT classifier when tested with six features. As shown in the figure, the classifier achieved an *FP* rate of 0.27% only, while maintaining an *FN* rate of 0.46%. These two rates are considered excellent in the area of intrusion detection, because it lowers the time, effort, and cost of handling false-positives, and minimizes the probability of false-negatives.

#### 6.3.2. 10-Fold Cross-Validation Results

The results of 10-fold cross-validation performed on RF classifier are shown in Table 3.

As shown in the table, the classifier maintained an accuracy of 0.996 to 0.997 throughout all of the folds, with an average of 0.9970703. In addition, the table shows minimal standard deviation in accuracy of 0.000224, and 0.000336 in $F_{1}$ Score. This means that the model would generalize well beyond its training dataset.

#### 6.3.3. Testing with IoT-ID and Aposemat IoT-23 Dataset

**Figure 4:**
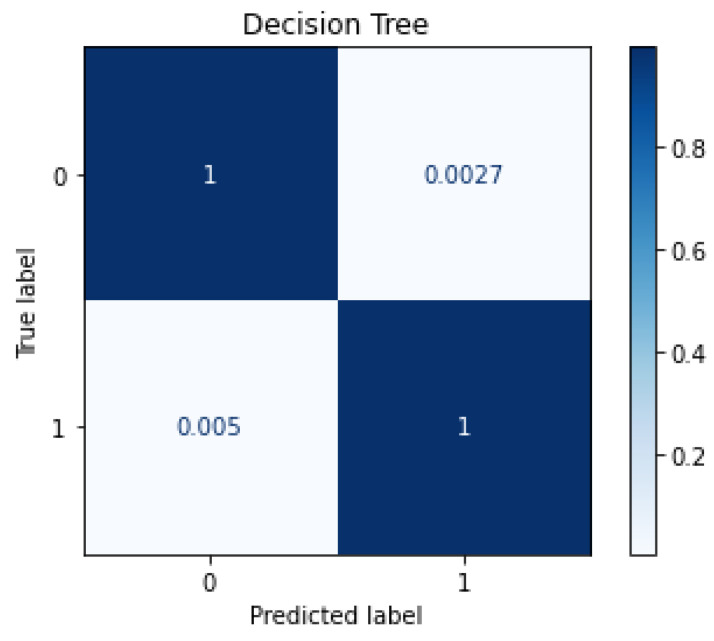
Confusion Matrix Plotfor Testing with IoT-ID Dataset.

The trained model was loaded and the IoT-ID dataset was used as input to it. Testing resulted in accuracy of 0.9963, and an average $F_{1}$ Score of 0.9962. Figure 4 shows the confusion matrix plot for the performance of the trained classifier when tested with the IoT-ID dataset. As shown in the figure, the classifier had a *FP* rate of 0.29%, and *FN* rate of 0.43% which is quite comparable to the *FP*, and *FN* rates of using TON_IoT dataset. The average testing time per network flow was 0.466 μs.

**Figure 5:**
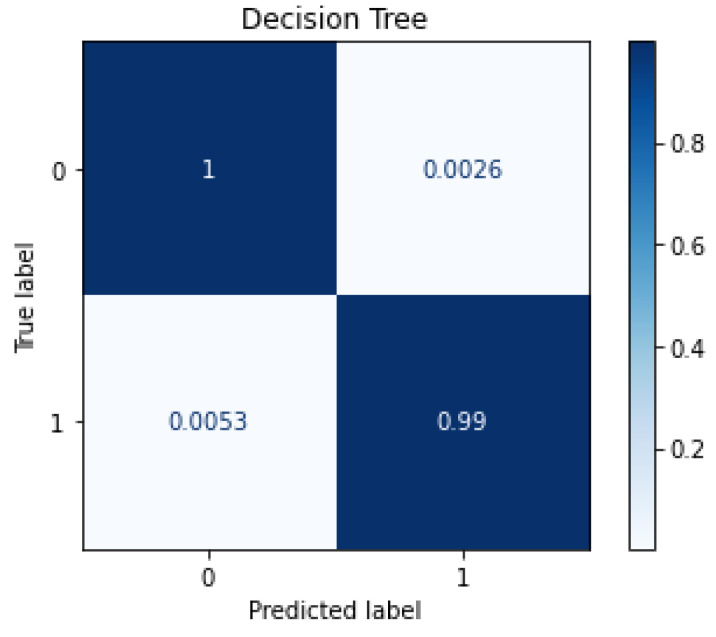
Confusion Matrix Plot for Testing with Aposemat IoT-23 Dataset.

When tested with with Aposemat IoT-23, the trained classifier produced an accuracy of 0.9961, with an average $F_{1}$ Score of 0.9961. Figure 5 shows the confusion matrix plot for the performance of the trained classifier when tested with the Aposemat IoT-23 dataset. As shown in the figure, the classifier had an *FP* rate of 0.27%, and an *FN* rate of 0.5% which is also comparable to rates generated by testing with TON_IoT dataset. The average testing time per network flow was 0.468 μs.

#### 6.3.4. Live Attack Testing

As a final validation step, we performed a port scan attack using nmap from a kali machine. The attack scenario was implemented by running the trained classifier on a virtual machine, and the attack was run from a separate kali linux offensive machine.

The command used to run the attack using the command nmap -p 1-1000 10.0.0.1, which initiates a port scan on 1000 ports. On the target machine, the packets were captured using the tcpdump tool in a pcap file, and the network flow features were extracted using Zeek. Then, the extracted features were fed into the trained classifier. The classifier captured all attack packets with an accuracy of 100%, and 0% false-negative rate.

## 7. Model’s Explainability

Explainability increases trust in the decisions made by the classifier. In addition, it prevents the classifier from operating as a black-box, and ensures that the high accuracy achieved by the classifier originates from explainable conditions [26].

Shapley additive explanations were used to explain how each of the selected features impacts the prediction of the trained model. SHAP was introduced in 2017 [27]. Its main strength over other explanation methods was that it is model-agnostic. The method is based on Shapley values introduced in game theory. Shapley values are found by calculating the impact of each player in a team by calculating the difference between the team’s performance with the player, and without the player. This helps in measuring the specific contribution of each individual player to the team’s performance. In explaining our proposed model, SHAP values measure the impact of each feature by measuring the model’s performance with the feature, and without it. This helps in understanding the impact of the feature on the prediction process. In our experiments, we used TreeExplainer as the SHAP explainer type.

**Figure 6:**
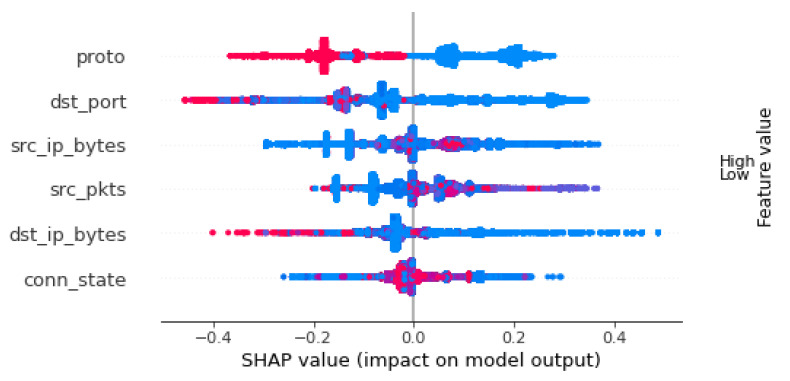
SHAP Values Summary Plot for the Selected Features.

Figure 6 shows the SHAP values summary plot of the selected six features. These six features are ordered in descending order from the feature with the highest impact on the decision to the lowest.

In Figure 6, the dots shown on the left side are the values that lower the prediction, which makes the prediction closer to “benign”, while the dots on the right side increases the prediction value which makes the prediction closer to “attack”. The dots in red are representative of a high value of the specific feature, while the blue dots are representative of a low value of the specific feature.

As shown in Figure 6, proto holds the highest impact on the predicted decision. The red dots on the left mean that the higher value of this feature would bring the prediction closer to “benign”. The values within this feature are: 1 for TCP, 2 for UDP, and 3 for ICMP. This explanation is consistent with the fact the most attacks utilize TCP protocol. This includes most common port scanning, HTTP flooding, SYN flooding, OS and host scanning. The blue dots on the right indicate that most attacks use TCP protocol at the transport layer.

The second feature in terms of the impact is dst_port. We can see that there are mixed dots on the left side of the plot. However, we can also see that very low port numbers are mostly indicative to an attack, with an exception of a cluster of blue dots between 0.0 and -0.2. This cluster indicates that a specific range of lower port-numbers are associated with legitimate traffic. In general, high port numbers push the prediction closer to “benign”. This explanation is generally consistent with the fact that most reconnaissance and brute force attacks in IoT context use lower port numbers as part of their scanning and logging-in activity.

According to Figure 6, the third feature in terms of impact is src_ip_bytes, which corresponds to the number of bytes within the flow of packets coming from the source IP address. The cluster of red dots on the right side means that there is a specific range of high number of bytes that would push the prediction towards “malicious”. The remaining range of blue dots makes it difficult to make a conclusive decision based on this feature only. This means that attacking traffic can have high or low number of bytes. This is consistent with the fact that some attacks require very low number of bytes, such as scanning attacks, while other attacks, such as flooding attacks, require a very high number of bytes. A similar explanation fits the src_pkts feature as well. A very high number of packets indicate a probable attack, while a low number of packets can be legitimate traffic, or an attack. This means that these two features need to be combined with other features to make a better decision.

The next feature in the figure is dst_ip_bytes. This feature represents the number of bytes sent from the responding host to the source of the flow. With the exception of the cluster of blue dots on the left side, a low number of response bytes mostly pushes the prediction toward “malicious”. As shown in Figure 6, high number of bytes, translating to larger meaningful response, is an indicator of “benign” traffic. This is consistent with the fact that the response triggered by attack packets is small, in general. For example, the response to an attempt to Telnet login to an IoT device is a small packet indicating that the login was denied. A similar case happens with scanning attacks, where the response is usually small. The cluster of blue dots on the left size indicates that there are benign flows that can have a low number of response bytes as well, but much smaller than the number of flows with high numbers of bytes in the benign category.

The feature with the least impact is conn_state. This field contains a code that describes the state of the connection at the end of t he flow. This field was encoded at the preprocessing phase. Hence, its values are of discrete meaning more than they are of continuous meaning. For example, the value S0, which means a connection without reply, was encoded as number 6. In Figure 6 we see a large cluster of red dots on the left side of the figure. This means that in most cases, a higher the value is, the more it’d push the prediction to benign. Most of normal uninterrupted flows has higher connection state values.

It is important to remember that the explanation detailed above should be considered as a collective explanation of the whole decision. Although the protocol feature holds the highest impact, it does not dictate the classifier’s outcome to be attack or benign traffic. It is the combination of the impact of all of the features that gives the classifier this high accuracy.

## 8. Discussion

### 8.1. Implementation Considerations

The selection of highly-effective features leads to models with improved implementability. This comes from two points; ensuring the ease of extraction of the selected features combined with minimal preprocessing requirements, and improving the prediction speed of the trained model by reducing the number of features needed to make the prediction, without sacrificing valuable accuracy.

Upon further examination the acquisition of the six selected features, we reached the following findings:

- dst_port: The destination port number can be easily extracted from a single packet without the need of waiting for the network flow to end or timeout;
- conn_state: This feature can be identified by various connection states, such as S0 (connection without reply), S1 (connection established), and REJ (connection attempt rejected). This information is collected from the TCP headers throughout the network flow;
- src_pkts: Number of original packets which is sent from source device. This information is calculated based on the whole packet flow;
- proto: The transport layer protocol of the flow connection. This feature can also be extracted from the first packet in the connection without the need to wait for the flow to end;
- src_ip_bytes: Number of origin IP bytes which is the total length of IP header field of source systems. This can be calculated from the captured packet flow;
- dst_ip_bytes: Number of destination packets which is estimated from destination systems. This can be calculated from the captured packet flow.

As shown above, three out of six of the selected features can easily be extracted from a single packet, while the remaining three require a complete capture of the network flow. To facilitate that in implementation, the model can be deployed on a network-border device, such as a firewall or a proxy, or in a host-based model. We recommend further study of the memory and storage requirements if deployed in a host-based model, which is beyond the cope of this work.

**Figure 7:**
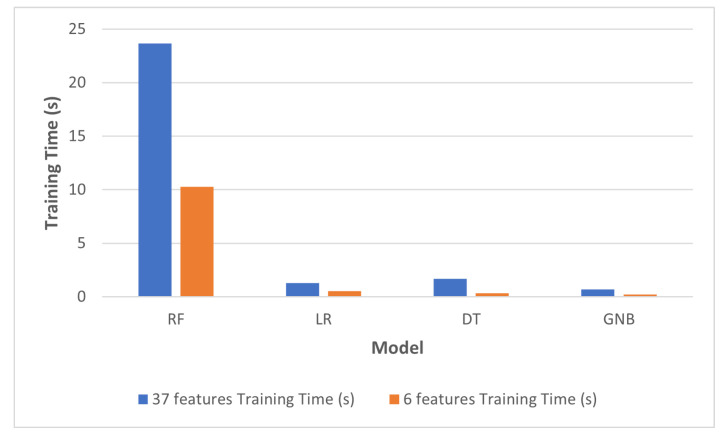
Change in training time after feature reduction.

**Figure 8:**
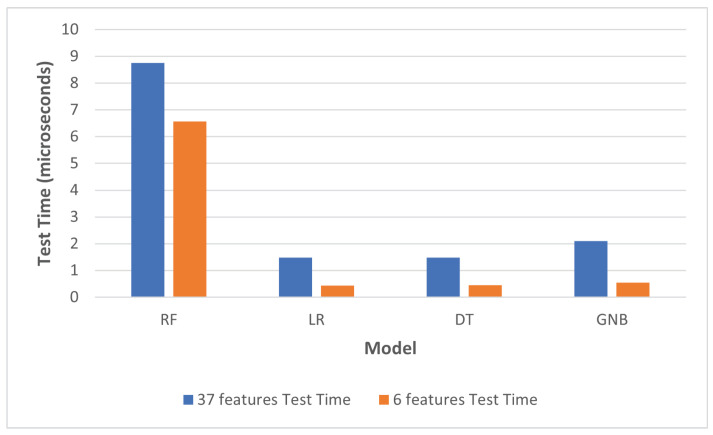
Change in testing time after feature reduction.

With regards to efficiency improvement, Table 4 shows the timing measures for all models utilizing the full 37-feature dataset, and with the reduced 6-feature dataset. The training time was measured for the complete subset used for training, while testing time was measured as an average time required for the prediction process per a single instance. The improvement in training and testing time can also be seen in Figure 7 and Figure 8, respectively.

According to Table 4, the reduction of training time achieved was 57%, 59%, 81%, and 70%, for RF, LR, DT, and GNB, respectively. This noticeable reduction in training time was due to the selection of a lower number of features.

On the other hand, per-instance testing time was reduced by 26%, 71%, 70%, and 21% for RF, LR, DT and GNB, respectively. This can have a significant impact on the performance of real-life deployments of the trained models.

### 8.2. Comparative Analysis

Table 5 shows a comparison with previous works, including papers [16,23,28,29]. These papers were selected because they relied on feature importance in performing feature selection.

With regards to timing parameters, our proposed system, especially the DT classifier, achieved the lowest prediction time with 0.45 μs when compared to the related works. This noticeable advantage is due to several reasons—the number of selected features is lower in comparison to the related works, and the use of classifiers that are less resources intensive such as DT, and RF. Classifiers that employ neural networks such as the ones used in [17,30] are considered more resource-intensive, and generally slower in producing predictions [24].

## 9. Conclusions and Future Work

We proposed in this paper an explainable efficiency- and implementation-focused universal feature selection for intrusion detection in IoT. The selected features were tested on three datasets—TON_IoT, IoT-ID, and Aposemat IoT-23—and produced a superior testing time and a very high accuracy exceeding 99% in detecting intrusions. The proposed feature selection method was based on recursive feature elimination based on feature importance measured in an RF classifier. The trained model went through three stages of testing; tested using 25% of TON_IoT dataset, 10-fold cross-validation, and tested using two more datasets (IoT-ID and Aposemat IoT-23).

Our proposed model was explained using SHAP values, and was found to be consistent with the known attack methodologies in terms of the selected features. The support of explainable machine learning increases trust in the proposed model, and ensures that its decisions are interpretable and do not originate from a blackbox. The explanation presented in Section 7 showed that the most effective feature in the decision making process was proto, while the least effective one was conn_state.

As future directions of this research work, the focus will be on the below listed points:

1. Measuring the performance of the trained model when deployed on border devices, such as firewall or proxy servers. This would help in having a better understanding of the practical requirements to make such systems operational;
2. Measuring the performance of the trained model when deployed on IoT devices and measure their processing requirements and performance. This would help in understanding deployment requirements for the proposed system as a host-based IDS;
3. Explore utilizing the reduced datasets in building deep neural networks. The utilization of deep neural networks can be explored in the context of network-based IDS to offset the processing load from the resource-constrained IoT devices to the border devices;
4. Improving the performance of Algorithm 2 to reduce the time required for the feature selection process.

## Figures and Tables

**Table 1:** Traffic included in the TON_IoT network-based train-test dataset.

Traffic Category	Number of Packets
Benign	300,000
Backdoors	20,000
DoS	20,000
DDoS	20,000
Injection	20,000
Password	20,000
Ransomware	20,000
Scanning	20,000
Cross-Site Scripting	20,000
MITM	1043

**Table 2:** Classifiers’ Performance Before and After Feature Selection.

	37 Features				6 Features			
**Model**	**Accuracy**	**$F_{1}$ Score**	**FP**	**FN**	**Accuracy**	**$F_{1}$ Score**	**FP**	**FN**
RF	0.9992	0.9992	0.0007	0.0006	0.9966	0.9966	0.0025	0.0042
LR	0.6502	0.3946	0.0000	0.0001	0.6500	0.3940	0.0000	1.0000
DT	0.9989	0.9988	0.0009	0.0013	0.9962	0.9962	0.0027	0.0046
GNB	0.6501	0.3947	0.0003	1.0000	0.4246	0.3381	0.8700	0.0300

**Table 3:** Results of 10-fold Cross-Validation Using DT Classifier.

Fold	Accuracy	Precision	Recall	$F_{1}$ Score
1	0.997289	0.995432	0.996848	0.996139
2	0.997245	0.995867	0.996297	0.996082
3	0.996920	0.995136	0.996005	0.995570
4	0.996681	0.995882	0.994579	0.995230
5	0.997245	0.995903	0.996212	0.996057
6	0.996855	0.995465	0.995402	0.995434
7	0.996877	0.995075	0.995944	0.995509
8	0.997397	0.995525	0.997013	0.996268
9	0.996942	0.995100	0.996259	0.995679
10	0.997180	0.995502	0.996484	0.995993
**Mean**	0.997063	0.995489	0.996104	0.995796
**S-Dev**	0.000224	0.000304	0.000667	0.000336

**Table 4:** Timing parameters of machine-learning models.

	37 Features		6 Features	
**Model**	**Train Time (s)**	**Test Time (μs)**	**Train Time (s)**	**Test Time (μs)**
RF	23.6616	8.7462	10.2907	6.5594
LR	1.3017	1.4834	0.5439	0.4346
DT	1.6723	1.4851	0.3339	0.4549
GNB	0.6859	2.0967	0.2129	0.5418

**Table 5:** Comparison of proposed system with related works.

Paper	Dataset	Features	Classifier	Accuracy (%)	Training T (s)	Testing T (s)
[15]	IoT-BoT	16	JRip	99.992	80.94	-
[17]	TON_IoT	44	CART	88	6.308	0.022
			RF	85	10.884	0.164
			KNN	84	58.018	109.361
			LSTM	81	1596	9.023
[30]	TON_IoT	13	ANN	84.39	-	-
	and		GBM	99.897	-	-
	Aposemat		RF	99.931	-	-
	IoT-23		MLP	99.022	-	-
[16]	TON_IoT	43	Extra Tree	97.86%	-	8.93 μs
Our work	TON_IoT	6	**DT**	**99.62**	0.3339	**0.4549 μs**
	IoT-ID	6	DT	99.63	0.3528	0.4663 μs
	Aposemat-IoT-23	6	DT	99.61	0.2973	0.4682 μs

## Data Availability

Not applicable..
